# The airway inflammation induced by nasal inoculation of PM2.5 and the treatment of bacterial lysates in rats

**DOI:** 10.1038/s41598-018-28156-9

**Published:** 2018-06-29

**Authors:** Yang Shen, Zhi-Hai Zhang, Di Hu, Xia Ke, Zheng Gu, Qi-Yuan Zou, Guo-Hua Hu, Shang-Hua Song, Hou-Yong Kang, Su-Ling Hong

**Affiliations:** 1 Department of Otorhinolaryngology, The First Affiliated Hospital of Chongqing Medical University, Chongqing, People’s Republic of China; 2 Chongqing Key Laboratory of Ophthalmology, Chongqing, People’s Republic of China

## Abstract

Particulate matter (PM) is one of the most important environmental issues in China. This study aimed to explore the correlation between PM2.5 and airway inflammation in healthy rats. The PM2.5 group was given an intranasal instillation of PM2.5 suspension on 15 consecutive days, and each received oral saline from day 16 to 90. The BV intervention group was treated as the PM2.5 exposure group, except that BV instead of saline was given daily. A histopathologic examination was performed to evaluate the airway inflammation. The prevalence and function of Th1/Th2/Treg/Th17 cells were detected by flow cytometry and ELISA. The expression of AhR was detected by western blot and real-time PCR. We found that epithelial damage and increased infiltration of inflammatory cell were present in the airways after PM2.5 exposure; there was an immune imbalance of Th cells in the PM2.5 group; the expression of AhR was increased in the airways after PM2.5 exposure. In the PM2.5 + BV group, we demonstrated alleviated immune imbalance and reduced inflammatory cell infiltration in the airways. Our study showed that exposure to PM2.5 induced airway inflammation. The imbalance of Th1/Th2/Treg/Th17 in PM2.5-induced airway inflammation might be associated with activation of the AhR pathway. Oral BV reduces PM2.5-induced airway inflammation and regulates systemic immune responses in rats.

## Introduction

Currently, particulate matter (PM) is one of the most important environmental issues in the modern society of China^1,2^. PM of various sizes is suspended in the atmosphere and contributes to ambient air pollution. PM2.5 comprises particulate matter with an aerodynamic diameter no more than 2.5 μm, which is the main component of air pollution in China^3^. PM2.5 poses a serious hazard to human health and deposits mainly in the tracheobronchial region. The toxicity caused by PM2.5 is a combined effect of particles and the adsorbed toxic pollutants, such as biological components (endotoxin, pollen, fungal spores, viruses, and bacteria), particles, polycyclic aromatic hydrocarbons (PAHs), volatile organic compounds (VOCs) and heavy metals^4–6^. It is well established that PM2.5 is more prone to attack the airways and circulation^7–9^. Research has shown that inhalation of PM2.5 is correlated with many airway diseases, such as allergic airway disease, acute airway inflammation, asthma and chronic obstructive pulmonary disease (COPD)^10–13^. Furthermore, it has adverse effects on lung function^14^. Recently, Keiki Ogino *et al*. reported that PM2.5 induces asthma-like airway inflammation in mice^10^, and the nasal inoculation of suspended PM2.5 induces inflammatory airway responses^15^. Moreover, Hetong Wang *et al*. found that short-term exposure to PM2.5 induces acute lung inflammation in Balb/c mice^11^.

The aryl hydrocarbon receptor (AhR) belongs to the basic helix-loop-helix-PER-ARNT-SIM family and is a ligand-dependent transcription factor that is involved in the detection of intracellular or environmental changes and sensing light, oxygen and redox potentials^16^. AhR is a multifunctional regulator that senses and responds to environmental stimuli and plays a role in normal cell development and immune regulation. It is known that dioxins and dioxin-like compounds, TCDD, PAH, and PM, can activate AhR, which then translocates to the nucleus and dimerizes with the AhR nuclear translocator (ARNT). Recent discoveries have demonstrated the interaction between AhR and environmental toxicants and its influence on immune responses^17–20^. Therefore, we hypothesized that AhR may help to modulate immune reactions in PM2.5-induced inflammation and function as a bridge between environmental factors and inflammatory airway diseases.

Broncho-Vaxom (BV), a bacterial lysate of 21 strains of 8 bacteria (Staphylococcus aureus, Haemophilus influenzae, Streptococcus pyogenes, Moraxella catarrhalis, Klebsiella pneumoniae, Klebsiella ozaenae, Streptococcus viridans and Diplococcus pneumoniae), has been shown to boost the immunological response^21^. Bacterial lysates have pleiotropic immunomodulating effects on both the innate and adaptive immune responses^22,23^. Research has shown that such lysates are able to induce an immunoregulatory response within mucosa-associated lymphoid tissue. This is associated with the suppression of Th2-induced immune responses, decreased allergen-specific IgE concentrations, and increased IL-10 secretion^24–26^. Recent studies have shown their efficacy for treating recurrent respiratory tract infections, asthma, chronic obstructive pulmonary disease (COPD), chronic bronchitis, subacute sinusitis, allergic rhinitis, chronic sinusitis and atopic dermatitis^25,27–31^. On the basis of these discoveries, we speculate that BV may be a novel approach for preventing and curing PM2.5-related inflammatory disease.

For a better understanding of the correlation between PM2.5 and airway inflammation, in this study, we aimed to evaluate the following hypotheses: (1) Exposure to PM2.5 from a traffic-related community would induce airway inflammation in normal healthy rats; (2) Inhaled PM2.5 may be involved in the pathogenesis of airway inflammatory disease through the AhR pathway; (3) BV may attenuate PM2.5-induced airway inflammation in rats by regulating immune disorders.

## Materials and Methods

### Animals

Rats were obtained from the animal experiment centre of Chongqing Medical University (Chongqing, China) at 6–8 weeks of age. All animals were housed in a specific pathogen-free environment. Housing and experimental conditions were maintained at 21–24 °C and 50–60% humidity. Light cycles were set on a 12-h light/dark cycle. The rats were provided with water and food ad libitum. The care and handling of the animals were in accordance with the Laboratory of Animals at Chongqing Medical University, which complies with the National Institutes of Health Guide for the Care and Use of Laboratory Animals. This study was approved by the Chongqing Medical University Institutional Animal Care and Use Committee.

### PM2.5 sampling and processing

Atmospheric PM2.5 samples were gathered using a Thermo Scientific TEOM1405-D particle monitor from March 14 to December 14, 2015, by the Chongqing Environmental Monitoring Centre. The sampling site was at the busy traffic intersection of the First Affiliated Hospital of Chongqing Medical University. PM2.5 was collected on filters, and the total mass was measured with a Tapered Element Oscillating Microbalance. Before being weighed, the glass fibre membrane was oven-dried at 60 °C for 6 h. Atmospheric PM2.5 at a busy traffic intersection was collected on the preweighed membrane. The negative pressures of the sampler during the PM2.5 collection ranged from −1.9 to −2.1 kPa. When the negative pressure increased up to −2.2 kPa, the membrane was replaced with a new one. The filters were dried (24 h at 60 °C) and weighed after PM collection. Filters containing PM2.5 were cut to 2 cm × 1 cm, which were then pooled in 100 ml deionized distilled water and administered by ultrasonic sonication for 2 h. The liquid containing the particulate matter was filtered through six layers of sterile gauze and centrifuged at 12,000 rpm at 4 °C for 30 min. Detached PM2.5 was then vacuum-freeze dried and weighed^32^. Particles were suspended in a certain amount of sterile saline to achieve PM2.5 suspensions with concentrations of 0.1, 0.3, and 0.5 mg/ml for the mouse model; the suspensions were stored at −20 °C.

### Administration to animals

Thirty rats were randomized into 3 groups: normal-control, PM2.5-exposure, and BV-intervention groups (N = 10/group). Controls were given an intranasal instillation of saline (20 ml/rat) on 15 consecutive days from day 1 to 15, and each received oral saline (0.2 ml/rat/day) from day 16 to 90. The PM2.5 exposure group was given an intranasal instillation of the PM2.5 suspension (0.3 mg/ml, 20 ml) on 15 consecutive days, and each received oral saline from day 16 to 90. The BV intervention group was treated as the PM2.5 exposure group, except that BV (25 mg in 500 µl of water) instead of saline was given daily. Animals were sacrificed 24 h after the last treatment.

### Histological analysis

Rat nasal and tracheal tissues were embedded in 4% paraformaldehyde, sectioned at 4–5 μm thicknesses and stained with haematoxylin and eosin (H-E). The histopathological lesions and changes were observed under a light microscope. The analyses were blindly accomplished by two independent researchers. Based on previous published methods^33,34^, the blinded histopathological score was used to assess the degree of nasal and bronchial inflammation on a subjective scale of 0–3, as follows: 0, no inflammatory cells; 1, occasional cuffing with inflammatory cells; 2, most bronchi or vessels surrounded by a thin layer of inflammatory cells of 1 to 5 cells thick; 3, inflammatory cell layer of more than five cells thick. The total airway inflammation was defined as the average of the nasal and bronchial inflammation scores.

### RNA extraction and real-time PCR analysis for AhR

Total RNA was extracted from lung tissue samples using TRIzol extraction (Invitrogen, Carlsbad, California, USA) according to the manufacturer’s instructions and was reverse-transcribed to cDNA with random hexamer primers and RNase H-reverse transcriptase (Invitrogen). The expression of mRNA was determined using the ABI Prism 7500 Sequence Detection System (Applied Biosystems, Foster City, California, USA) and SYBR Premix Taq (TaKaRa Biotechnology, Dalian, China). The following primer pairs were used for AhR: F: 5′-TCCCTTATGAGTGCCTTGA-3′, R: 5′-GTCTGATTTCCTCGTGTTTC-3′. All PCR reactions were performed in duplicate according to the following program: 50 °C for 2 min, 90 °C for 10 min, 50 cycles of 95 °C for 15 s, and 60 °C for 1 min. Relative gene expression was calculated by using the comparative CT method. β-actin was used as a housekeeping gene for normalization, and a no-template sample was used as a negative control.

### ELISA analysis for cytokines

Serum was collected and stored at –80 °C. The levels of IFN-γ, IL-4, IL-5, IL-10, IL-13 and IL-17 in the supernatants were assayed using specific ELISA kits according to the manufacturer’s instructions (all ELISA kits from eBioscience, San Diego, CA). All assays were performed in duplicate. The results are expressed in pg/ml.

### Cell preparation and flow cytometric analysis for Th1/Th2/Treg/Th17 cells

Spleen tissues were collected, cut into small fragments, and teased apart to allow dispersion of the cells into RPMI 1640. The cells were passed through a 40 μm mesh to obtain a single cell suspension. Following a rinse, the cells were adjusted maximally to 2 × 10^6^ cells/ml. For Th1, Th2 and Th17 detection, the cells were activated with phorbol myristate acetate (PMA, 50 ng/ml; Alexis Biochemicals, San Diego, CA) and ionomycin(1 μM; Sigma, USA) in the presence of monensin (1 μl/ml; BD, USA) for 4 h at 37 °C in a 5% CO_2_ atmosphere. For the analysis of Treg, cells were aliquoted into tubes for further staining.

We defined Th1 as CD3^+^CD4^+^IFN-γ^+^ cells, Th2 as CD3^+^CD4^+^IL-4^+^ cells, Treg as CD4^+^CD25^+^Foxp3^+^ cells, and Th17 cells as CD3^+^CD4^+^IL-17^+^ cells. The cells were incubated with PE-conjugated anti-rat CD3 and FITC-conjugated anti-rat CD4 for Th1, Th2, and Th17 analysis. The cells were incubated with FITC-conjugated anti-rat CD4 and PE-conjugated anti-rat CD25 for Treg analysis. After surface staining, cells were re-suspended in a fixation and permeabilization solution according to the manufacturer’s instructions and then stained with Anti-rat IFN-gamma eFluor® 660/Anti-rat IL-4 eFluor® 660/Anti-rat Foxp3 APC/Anti-rat IL-17A APC. All of the antibodies were from eBioscience. Fluorescence profiles were analysed using a FACScan cytometer equipped with CellQuest software (BD). The results are expressed as a percentage of positive cells.

### Western blotting analysis for AhR

Lung tissue was lysed with a radio-immunoprecipitation assay (RIPA) lysis buffer (containing 1% phenylmethylsulfonyl fluoride). The protein concentrations of the extracts were measured with a protein assay 10 kit (Beyotime, China). Equal amounts of protein were loaded and separated on sodium dodecyl sulfate–polyacrylamide gels (AhR: 6% gel; β-actin: 10% gel), transferred onto polyvinylidene difluoride membranes and incubated with anti-AhR (Sigma-15 Aldrich, USA) or anti-β-actin (Beyotime) primary antibodies. The immunoreactivity of proteins in the membrane was determined using an ECL chemiluminescence reaction kit, followed by exposure to a medical film according to the manufacturer’s instructions. Quantity One software, version 4.52 (Bio-Rad) was used to quantify the relative expression levels of the proteins.

### Statistical analysis

The software used for statistical analysis was SPSS for Windows ver. 17.0 (SPSS, Chicago, IL). Data are presented as mean ± standard deviation or medians and interquartile ranges. Differences between the values were determined using Student’s t test. Grouped data were analyzed using a one-way analysis of variance (ANOVA) followed by the Student–Newman–Keuls test. When the equal variance test failed, a Mann–Whitney Rank Sum test was used. Significance was accepted at P < 0.05.

## Results

### Effect of PM2.5 on airway inflammation

**Figure 1:**
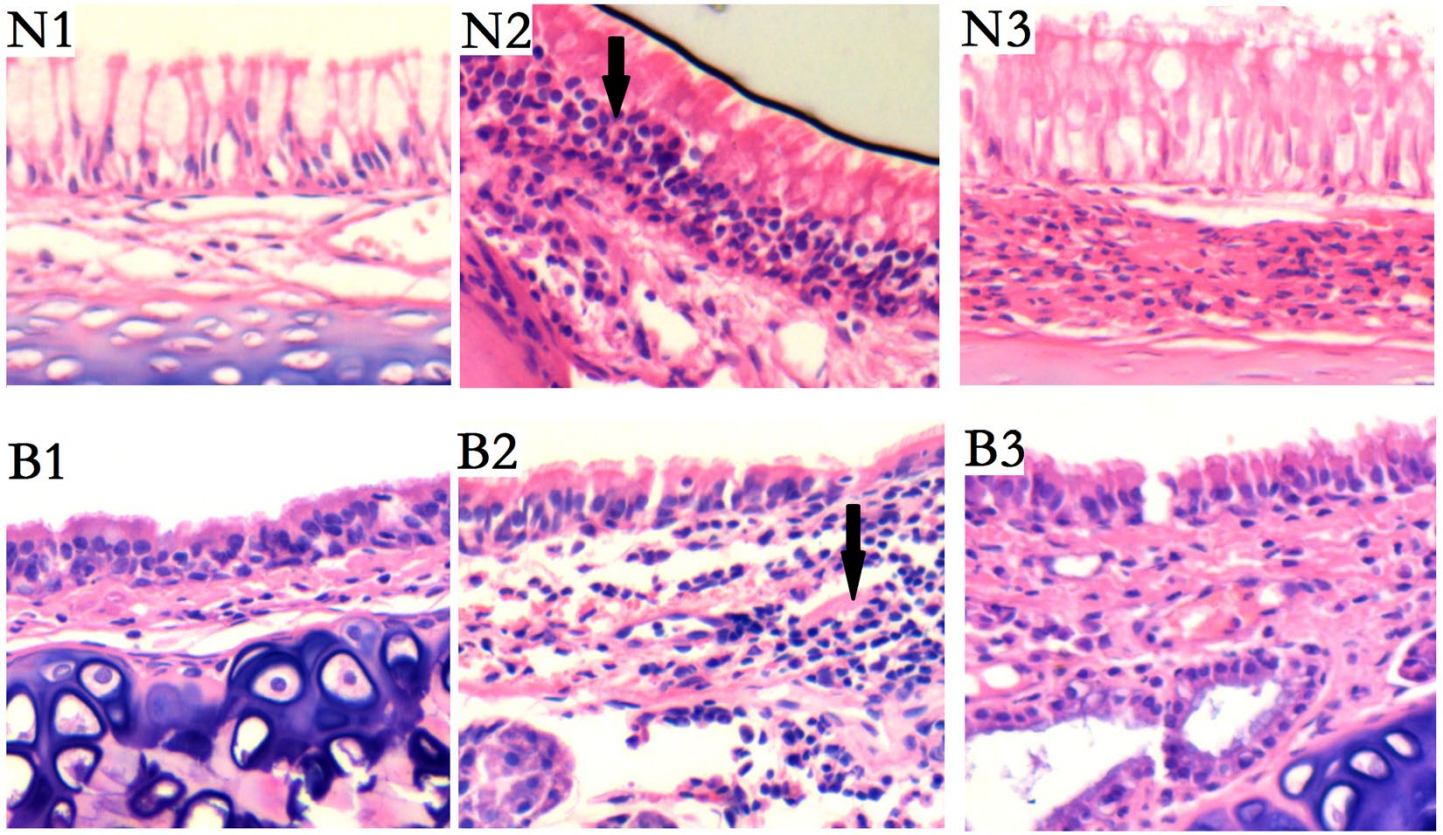
Effects of PM2.5 on recruitment of inflammatory cells to airway. Representative airway sections were H&E stained to estimate inflammation (black arrows) in nasal and tracheal regions of each group (N1–3: nasal mucosa in control, PM2.5, PM2.5 + BV group; B1–3: bronchial mucosa in control, PM2.5, PM2.5 + BV group; magnification 200x).

**Figure 2:**
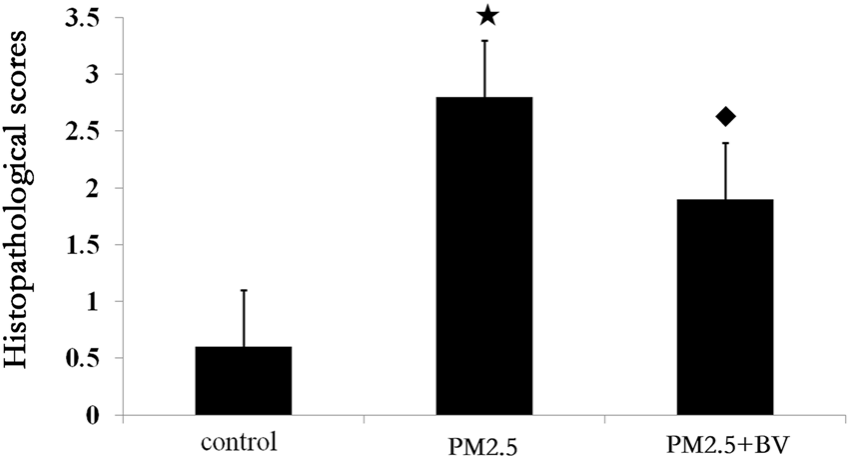
Histopathological scores of airway in in control, PM2.5, PM2.5 + BV group, ^★^P < 0.01, vs. control; ^◆^P < 0.01, vs. PM2.5 group.

To evaluate PM2.5-induced airway inflammation in rats, a histopathologic examination was performed. As shown in Fig. 1, the PM2.5 and PM2.5 + BV groups showed significantly increased airway infiltration of inflammatory cells compared with the control groups. Histopathological scoring was used to evaluate the nasal and bronchial inflammatory cell recruitment in each group (Fig. 2).

### Effect of PM2.5 on the Th1/Th2/Treg/Th17 balance analysis

**Figure 3:**
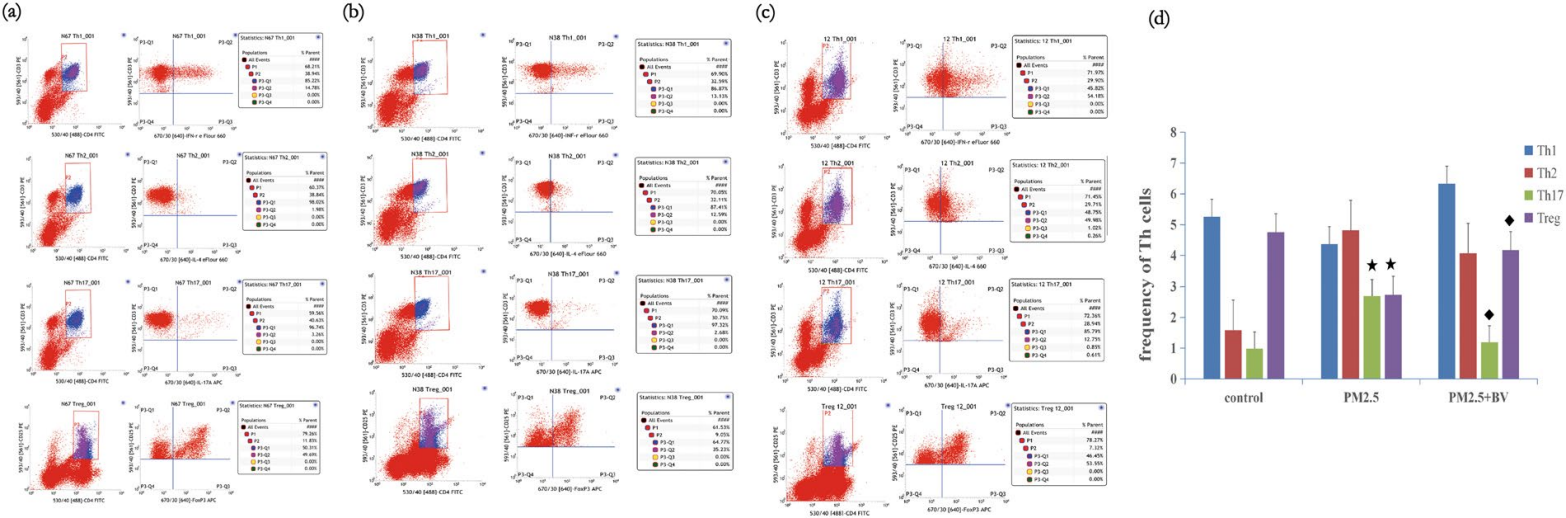
Flow cytometric analysis of Th1/Th2/Treg/Th17 cells in spleen tissue of control (**a**), PM2.5 group (**b**) and PM2.5 + BV rats (**c**). (**d**) The frequency of Th1/Th2/Treg/Th17 cells. ^★^P < 0.01, vs. control; ^◆^P < 0.01, vs. PM2.5 group.

The prevalence of Th1, Th2, Treg and Th17 cells in spleen cells were detected by flow cytometry. Our results showed that the frequencies of Th17 cells were significantly upregulated in PM2.5-induced rats compared with those in the control group (2.694 ± 1.480, 0.986 ± 0.401, P = 0.021). In addition, the frequencies of Treg cells were significantly downregulated (4.754 ± 1.421, 2.734 ± 0.755, P = 0.07). However, there were no significant differences in Th1 and Th2 cells between PM2.5-induced rats and controls, although there was an increasing trend in the frequency of Th2 cells (4.362 ± 2.392, 5.253 ± 1.873, P = 0.483; 4.816 ± 3.453, 1.582 ± 0.798, P = 0.055) (Fig. 3).

### Effect of PM2.5 on the expression of Th cell-related cytokines

**Figure 4:**
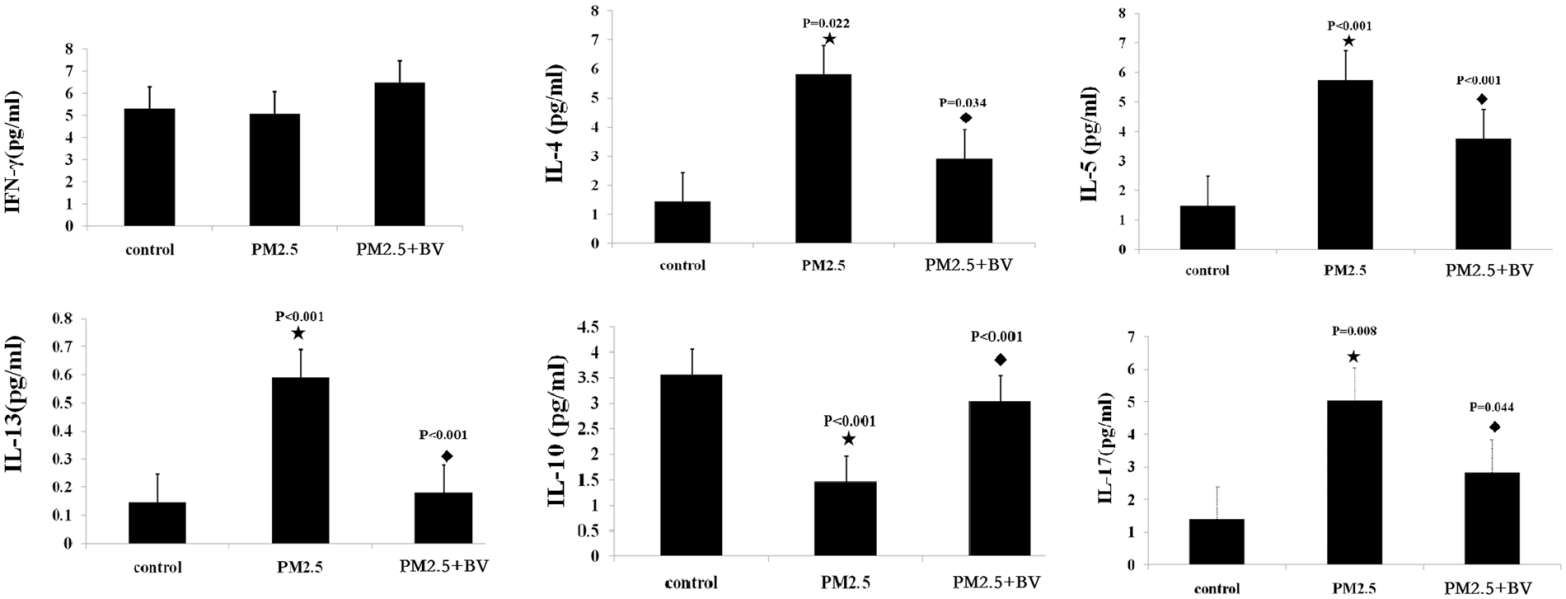
The levels of IFN-γ (**a**), IL-4 (**b**), IL-5 (**c**), IL-13 (**d**), IL-10 (**e**), and IL-17 (**f**) in serum were detected by ELISA. ^★^P < 0.01, vs. control; ^◆^P < 0.01, vs. PM2.5 group.

The levels of related cytokines for Th1, Th2, Treg and Th17 were assessed using ELISA tests. Consistent with the results of flow cytometry, IL-4, IL-5, IL-13, and IL-17 levels were significantly increased in PM2.5-induced rats (5.820 ± 0.848, 5.743 ± 0.535, 0.597 ± 0.012, 5.044 ± 1.378; P = 0.022, P < 0.001, P < 0.001, P = 0.008) versus controls (1.438 ± 0.049, 1.483 ± 0.311, 0.146 ± 0.010, 1.388 ± 1.103), while IL-10 levels were significantly decreased in PM2.5-induced rats (1.467 ± 0.096, 3.562 ± 0.116, P < 0.001). Meanwhile, there were no significant differences in IFN-γ levels between PM2.5-induced rats (5.063 ± 0.390, P = 0.056) and controls (5.288 ± 0.614) (Fig. 4).

### Effect of PM2.5 on AhR expression

**Figure 5:**
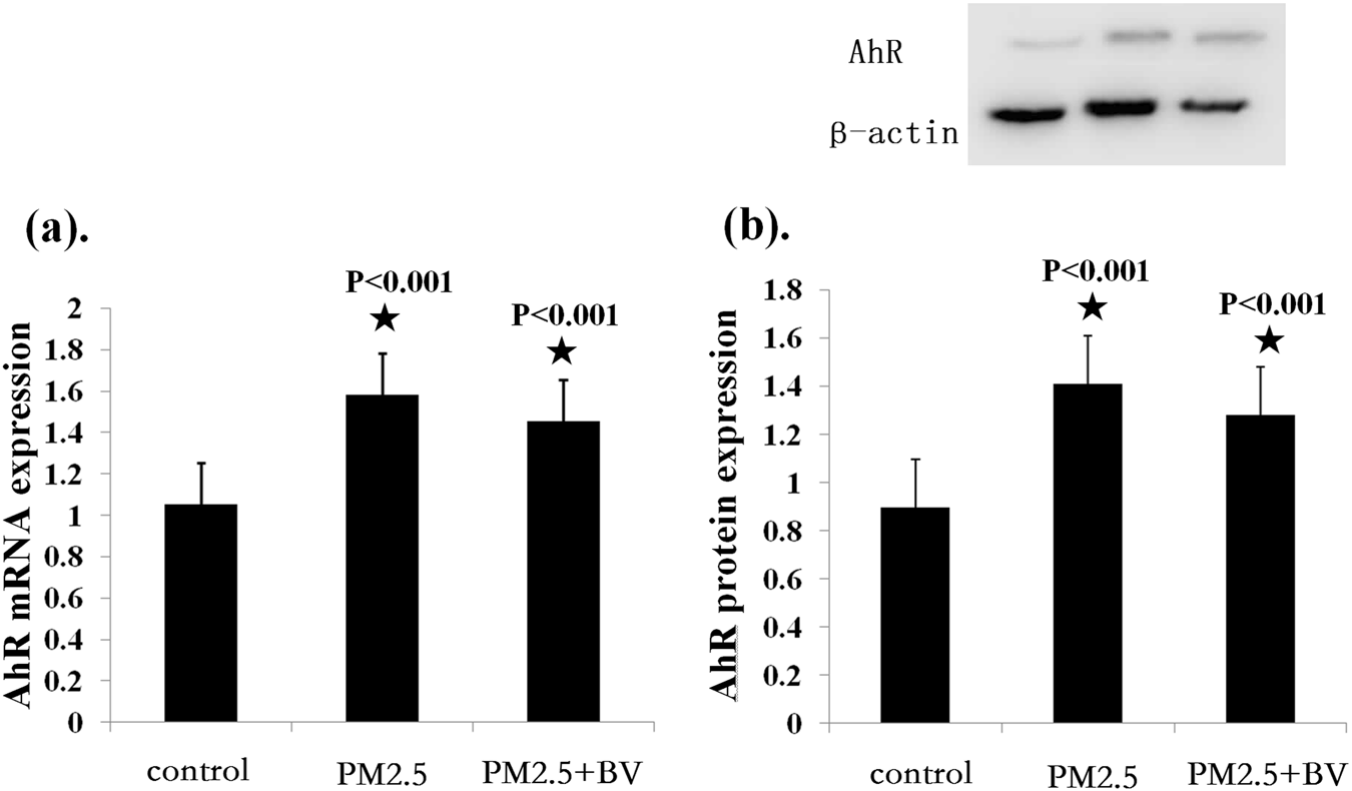
Real-time PCR and western blot analysis of AhR in lung tissue. (**a**) The ratios of AhR/β-actin mRNA were compared among groups; (**b**) The ratios of AhR/β-actin protein were compared among groups. ^★^P < 0.01, vs. control.

Lung tissue from PM2.5-exposed rats, BV intervention rats, and controls was used to assay the mRNA and protein expression of AhR. The results showed that AhR mRNA expression was significantly increased in PM2.5-exposed rats compared with controls (1.583 ± 0.091, 1.055 ± 0.102, P < 0.001). Meanwhile, AhR protein expression was also significantly increased in lung tissue from PM2.5-exposed rats compared with those from controls (1.410 ± 0.039, 0.900 ± 0.083, P < 0.001). However, there were no significant differences concerning AhR mRNA and protein expression between PM2.5-exposed rats and BV intervention rats (1.455 ± 0.093, P = 0.55; 1.282 ± 0.066, P = 0.053) (Fig. 5).

On the basis of our previous study results, which demonstrated that upregulation of AhR might be associated with the inflammatory response in AR patients and that the nontoxic AhR ligand ITE significantly reduced the inflammatory response by inhibiting Th17 cell differentiation and downregulating the production of IL-17, we speculated that PM2.5 may induce airway inflammation and impair Th balance through AhR pathway.

### Effect of BV on alleviating PM2.5 induced airway inflammation

To investigate the functions of BV in PM2.5-induced airway inflammation, we administered BV to mice after PM2.5 exposure. Lung and spleen tissues, airway mucosa and serum samples were collected. The histopathological changes in the airway were observed by H&E staining. According to the histopathological result, BV alleviated the PM2.5-induced airway inflammation. The PM2.5 + BV groups showed increased airway infiltration of inflammatory cells. However, the inflammatory cell infiltration in the PM2.5 + BV groups was statistically decreased compared with that of the PM2.5 group (Figs 1 and 2).

### Effect of BV on modulating Th immune responses in PM2.5 induced airway inflammation

To investigate the functions of BV in reducing PM2.5-induced airway inflammation, we detected the prevalence of Th cells in rat spleen tissues and related effector cytokines in the serum. According to the results, the frequency of Th17 cells in the PM2.5 + BV group was downregulated compared with that in the PM2.5 group (1.187 ± 0.694, P = 0.44), and the frequency of Treg cells in the PM2.5 + BV group was significantly upregulated (4.172 ± 0.755, P = 0.007). In addition, the frequency of Th2 cells in PM2.5 + BV group was downregulated, but the differences were not significant (4.070 ± 4.103, P = 0.909) (Fig. 3).

Furthermore, our data showed that the IL-4, IL-5, IL-13, and IL-17 protein expression levels in the sera of the PM2.5 + BV group were significantly decreased compared with those of PM2.5 induced rats (2.916 ± 0.310, 3.750 ± 0.380, 0.179 ± 0.027, 2.667 ± 0.895; P = 0.034, P < 0.001, P = 0.044). Meanwhile, the IL-10 protein expression level in the sera of the PM2.5 + BV group was significantly increased (3.051 ± 0.254, P < 0.001). The results suggested that BV decreases the inflammatory response by modulating Th immune responses in rats exposed to PM2.5 (Fig. 4).

## Discussion

In this study, our findings can be summarized as follows: (1) The data showed epithelial damage and increased infiltration of inflammatory cells in the airway after PM2.5 exposure, suggesting that PM2.5 from a traffic-related community can induce airway inflammation in normal healthy rats; (2) There was an immune imbalance of Th1/Th2/Treg/Th17 cells in PM2.5-induced rats; (3) AhR, which responds to environmental stimuli and plays a role in immune regulation, was found to have an increased expression in the airway after PM2.5 exposure, suggesting that inhaled PM2.5 may be involved in the pathogenesis of airway inflammatory disease through the AhR pathway; (4) In the PM2.5 + BV group, we demonstrated alleviated immune imbalance and reduced inflammatory cell infiltration in the airway, suggesting that a bacterial lysate may attenuate PM2.5-induced airway inflammation in the rat by regulating immune disorders. To the best of our knowledge, the present study is the first to explore the effect of oral BV on PM2.5-exposure induced airway inflammation.

First, we examined the initiation of PM2.5-induced airway inflammation. After PM2.5 exposure, inflammatory cell infiltration into the airway was readily observed. Furthermore, the imbalance of CD4^+^ T-cell subsets, which include Th1, Th2, Th17 and Treg cells, has been implicated in the aetiology of PM2.5-induced airway inflammation. The prevalence and function of Th17 cells were significantly upregulated, while Treg cells were downregulated. Our results are mostly consistent with previous reports in which the characteristics of a PM2.5-related inflammatory airway response and immune disorder were noted^10,15^. IL-17A, which plays an important role during the pathophysiological process of allergic airway inflammation, especially in neutrophilic inflammation^35,36^, exhibited increased expression in PM2.5-induced airway inflammation. Moreover, Th17 cells, a subset of Th cells, have been considered the primary producer of IL-17A^37,38^. The frequency of Th17 cells was significantly increased. These data suggested that the increased IL-17A and Th17 cells attributed to PM 2.5 exposure and played a vital role in PM2.5-induced airway inflammation.

Our recent research demonstrated that in AR, the nontoxic AhR ligand ITE significantly reduced the inflammatory response by inhibiting Th17 cell differentiation and downregulating the production of IL-17^39^. To explore the further pathogenesis of PM2.5-induced airway inflammation, we focused on the role of AhR in the pathology process. AhR is a multifunctional regulator that senses and responds to environmental stimuli and plays a role in normal cell development and immune regulation. It has been considered the primary receptor or sensor for various pollutants that are present in automobile exhaust, industrial waste, and cigarette smoke^40^. It is known that dioxins and dioxin-like compounds, TCDD, PAH and particulate matter (PM), can activate AhR. Recent discoveries have demonstrated the interaction between AhR and environmental toxicant interaction and its influence on immune responses^17–20^. Meanwhile, AhR has been found to be highly expressed on dendritic cells, Th17 cells, and other immune cells^40^. However, to the best of our knowledge, the expression of AhR on the rat airway mucosa has not been previously addressed. Interestingly, in this study, we found that AhR was constitutively expressed in the normal rat airway mucosa and upregulated in the PM2.5-exposed mucosa, where it was mainly distributed in the superficial epithelium, submucosal glands, vascular endothelium, and inflammatory cells that had infiltrated into the mucosa. Furthermore, the mRNA and protein expression of AhR in lung tissue was significantly increased in PM2.5-exposed rats. On the basis of our results and recent discoveries, we speculate that AhR functions as a bridge between PM2.5 pollution and inflammatory airway diseases. PM2.5 may induce airway inflammation and impaired Th balance through the AhR pathway.

BV, which is used to treat recurrent respiratory tract infections, asthma, COPD, chronic bronchitis, allergic rhinitis, chronic sinusitis and other respiratory diseases, is able to induce an immunoregulatory response within mucosa-associated lymphoid tissue. In murine models of asthma, CD4^+^ T-helper cells in the lungs and bronchoalveolar lavage fluid decrease eosinophilia and the Th2 cytokine secretion of IL-4, IL-5, IL-13, and TGF-β. In addition, the oral administration of bacterial lysates increases the CD4^+^CD25^+^Foxp3^+^ regulatory T-cell population in airway mucosal tissue and attenuates airway hyperresponsiveness^26,41^. In support of these findings, our study demonstrated that the administration of BV induced Treg cells and suppressed Th17 activity in PM2.5-induced airway inflammation, which provides a new target for the treatment of PM2.5-related airway inflammatory diseases.

Our study had certain limitations. We did not determine the chemical components of PM2.5. The mean daily concentration of PM2.5 in Chongqing was recently reported to be 147 mg/m^3^ (maximum, 666 mg/m^3^). Furthermore, a rapidly growing body of literature has characterized the components of PM2.5 in traffic-related air pollution^33,42–44^. Our PM2.5 was collected near a main road in Chongqing, and the components would be mainly consistent with those of the traffic-related PM2.5 reported in previous research. Thus, we did not analyse them. In addition, our data revealed that AhR expression was significantly increased in lung tissue from PM2.5-exposed rats and that PM2.5 might induce airway inflammation and impaired Th balance through the AhR pathway. However, further intensive investigations are required to elucidate the specific mechanism of the AhR pathway. For example, the AhR signalling pathway and the role of ligands for AhR would be interesting to pursue in our future research.
